# Participation and quality of life of Nepalese children with visual impairment in comparison with normally sighted peers: a cross sectional comparative study

**DOI:** 10.1186/s41687-025-00893-2

**Published:** 2025-06-05

**Authors:** Srijana Adhikari, Ellen Bernadette Maria Elsman, Ruth Marie Antoinette van Nispen, Fleur Van Rens, Manish Poudel, Gerardus Hermanus Maria Bartholomeus van Rens

**Affiliations:** 1 Tilganga Institute of Ophthalmology, Gaushala Kathmandu, Nepal; 2 Department of Ophthalmology, VU University Medical Centre and the Amsterdam Public Health Research Institute, PO Box 7057, Amsterdam, 1007 MB The Netherlands; 3 Amsterdam Public Health, Quality of Care, Amsterdam, The Netherlands; 4 School of Allied Health (Exercise Science), Murdoch University, Murdoch, Australia

**Keywords:** Participation and activity, Children, Visual impairment, Blindness, Quality of life, PROMs

## Abstract

**Background:**

Poor vision compromises quality of life and participation in different daily life activities of children such as, sports, leisure time, interactive play and social interaction. The purpose of this cross - sectional study is to investigate participation and quality of life of children with visual impairment (VI) and blindness compared with normally sighted peers.

**Methodology:**

Children aged 7–17 years with blindness (*n* = 100), moderate to severe VI (*n* = 100) and normal sight (*n* = 100) completed Nepalese versions of the Participation and activity inventory children and youth (PAI-CY 7–12 and 13–17), L. V. Prasad functional vision questionnaires (LVP-FVQ II) and Pediatric Eye Questionnaires (PedEyeQ 5–11 and 12–17). The measurement properties of PAI -CY was studied. All (sub) scores were compared between three groups. Associations between the severity of VI and outcomes were assessed with age and sex adjusted linear regression analyses.

**Results:**

Children with blindness scored worse than children with VI, who scored worse than normally sighted children on the PAI-CY 7–12, the physical functioning subscale of the PAI-CY 13–17 and the LVP-FVQ II(p = < 0.001).However, for the psychosocial functioning subscale of PAI-CY 13–17, children with blindness scored better than children with VI(p = < 0.01). On the PedEyeQ, young children (5–11) with blindness on all subscales, and older children (12–17 years) with blindness on the functional vision subscale scored worse than children with VI(p = < 0.01).Regression models showed that both moderate/severe VI and blindness were significantly associated with worse PAI-CY, LVP-FVQ II, PedEyeQ 5–11 and PedEyeQ 12–17 functional vision subscale scores(p = < 0.01).

**Conclusion:**

Younger children with blindness showed worse participation and quality of life compared to children with VI, whereas results for older children showed a mixed pattern with children with blindness showing better participation in psychosocial domain. Appropriate low vision rehabilitation interventions are needed for children with VI and blindness to increase their participation and quality of life to the level of their normally sighted peers as far as possible. Future studies could include children who do not attend school and may have worse participation and quality of life than children in our study.

**Supplementary Information:**

The online version contains supplementary material available at 10.1186/s41687-025-00893-2.

## Background

Visual impairment (VI) is one of the most common disabilities in children worldwide [1]. The prevalence of blindness in children around the globe ranges from 0.3/1000 children in high- income countries to 1.5/1000 children in the least developed countries [2]. Studies conducted in Nepal have shown that the prevalence of childhood blindness ranged between 0.03 and 0.06% [3, 4]. Participation is defined as a person’s “involvement in a life situation” and represents the societal perspective of functioning [5, 6]. In 2007 WHO introduced the International Classification of Functionality and disability for children and Youth (ICF-CY) [7]. The ICF-CY has given special attention to participation as life situations in children differ significantly from those of adults. Optimal visual input is required for the development of cognitive and functional skills in children [8]. VI in children is associated with poor motor development, social and problem-solving skills [9–12]. Apart from this, poor vision compromises quality of life and participation in different daily life activities such as social and romantic interaction, sports, leisure time and interactive play [13–17]. Similarly, children with VI face challenges related to activities and participation [18–19]. Clinical examination of children with VI including an assessment of visual functioning is helpful to identify the extent of the experienced visual disability which in turn is necessary in planning and monitoring adequate rehabilitation interventions [20, 21]. On the other hand, assessment of quality of life and participation in different life situations of these children provides a broader picture of the impact of VI on the lives of these children and their families. This may help to provide rehabilitation services and personalized care [17, 18, 22, 23]. Measuring quality of life and participation in children and adolescents with VI can be considered important throughout the course of their development. Children with VI are the best judges of their own activity limitations, participation, and quality of life. In recent years patient-reported outcome measures (PROMs) have been developed specifically for children with VI. Most PROMs focus on vision-related quality of life or functional vision which refers to how well an individual performs while interacting with the visual environment [24–29]. For example, the LV Prasad Functional Vision Questionnaire (LVP-FVQ) was the designed to assess functional vision in children with VI in developing countries [30, 31], whereas the Pediatric Eye Questionnaire (PedEyeQ) was developed to measure functional vision and eye-related quality of life in children with any eye condition [32]. Moreover, the Participation and Activity Inventory for Children and Youth (PAI-CY) consists of a series of PROMs to measure limitations in activities and participation in children with VI in different age groups from 0 to 17 years. The PAI-CY was originally developed in a Dutch pediatric population [33–38].

Although a handful of studies is available from neighboring countries like China, the Philippines and India [23, 39, 40], little is known about participation and quality of life of children with VI in Nepal. Moreover, there are very few studies in literature which compares quality of life and participation of children with VI or normally sighted children [41]. The aim of the current study was (1) to investigate limitations in activities and participation, and quality of life of children with VI and to compare these outcomes with normally sighted peers and (2) to evaluate the association between the severity of vision loss and these important health outcomes. We hypothesize that children with VI have worse participation and quality of life than the normally sighted children and that there is an association between severity of VI and participation and quality of life outcomes.

## Methods

### Study design

This cross-sectional study is part of the Nepal Pediatric Vision Impairment Study (NPVIS) which aims to study activity, participation, sleep and quality of life of children with VI compared to normally sighted peers. The study was carried out in five integrated schools for the blind in and around the Kathmandu vally and the Tilganga Institute of Ophthalmology over a period of 14 months, from April 2021 to June 2022. An observation visit was carried out to estimate the number of children with blindness studying in the integrated schools for the blind in and around Kathmandu valley. Through purposive sampling, children with blindness (*N* = 100) and normally sighted children (*N* = 100) were recruited from the integrated schools for the blind. Children with moderate and severe VI (*N* = 100) were recruited through consecutive sampling from the outpatient department of the Tilganga Institute of Ophthalmology in Kathmandu. A sample size of 100 for each group is considered “very good” by standards of the COSMIN initiative [42]. The study was approved by the Ethical Review Board of the Nepal Health Research Council (239–2020). The study adhered to the tenants of the Declaration of Helsinki. The approval letter from each school selected for the study were approved by the school administration. Informed consent was provided by the parents of the children included in the study. Ascent was provided by the children themselves who were between the age of 12 to 17 years.

### Participants

Participants included children aged between 7 and 17 years. Children with blindness (best corrected visual acuity (BCVA) < 3/60 in the better eye), were age- and sex-matched to normally sighted peers (BCVA ≥ 6/12) from the same school and grade level. Participants with moderate (BCVA < 6/12 to 6/60) and severe (BCVA < 6/60 to 3/60) VI were those who visited the outpatient department of the Tilganga Institute of Ophthalmology in Kathmandu during the study period. Children with developmental delay, cognitive impairment or other disabilities apart from VI were excluded from the study.

### Procedure

Demographic information including age, sex and living condition (hostel vs. day scholar) was collected. Each child underwent a vision assessment which consisted of visual acuity examination. Children with VI additionally underwent anterior and posterior segment evaluation. All children completed PROMs through face-to-face interviews by a community health worker, which were audio recorded for later verification. A repeat interview to assess test-retest reliability was carried out 2 weeks following the initial interview. PROMs included the Nepalese versions of the PAI-CY 7–12, or 13–17, depending on the age of the child, and the Nepalese version of the LVP-FVQ II and PedEyeQ 5–11 or 12–17. Table 1 details which PROMs were completed by which subgroups.

**Table 1:** PROMs completed by different subgroups

PROM	Normally sighted (*N*)	Moderate / severe VI (*N*)	Blind (*N*)
PAI-CY (7–12)	50	68	47
PAI-CY (13–17)	50	32	53
LVP-FVQ II (7–17)	100	100	100
PedEyeQ (5–11)	0	58	35
PedEyeQ (12–17)	0	41	65

### PROMs

The Nepalese version of the PAI-CY 7–12 consists of 61 items which cover nine domains (for descriptive purposes only): play, social contact, mobility, leisure time, communication, school, self-reliance, acceptance/self-consciousness and finance. The Nepalese version of PAI-CY 13–17 consists of 68 items which cover eight domains: leisure time, mobility, social contact, communication, school, self-reliance, acceptance/self-consciousness and finance. Responses to the items on both versions are provided on a four-point Likert scale: (1) not difficult; (2) slightly difficult; (3) very difficult; and (4) impossible. The response option ‘not applicable’ was treated as missing value.

The LVP-FVQ II contains 23 items which are answered on a four-point Likert type scale: (1) no difficulty; (2) some difficulty; and (3) a lot of difficulty. The questions are designed to cover four domains: distance vision, near vision, color vision and visual field, but a total score is calculated. It was developed for children 8–17 years, however, we used it in children between 7 and 17 years.

The child versions of the PedEyeQ 5–11 and 12–17 were used, depending on the age of the child. Both versions consist of four domains with 10 items in each domain: functional vision, bothered by eyes/vision, social and frustration/worry. Responses to the items are provided on a three-point Likert scale: (2) never; (1) sometimes; and (0) all of the time.

### Measurement properties of proms

Previously we culturally adapted the PAI-CY for use in Nepal [43] and therefore some measurement properties were studied. First, item analyses for the two age versions of the PAI-CY were performed to investigate floor and ceiling effects at the item level and distribution over the response categories. Items with > 50% missing responses were deleted. Items with an inter-item correlation > 0.7 were flagged for potential redundancy. Next, structural validity was first investigated through principal component analysis (PCA). The number of factors was examined through a combination of visually inspecting the scree plots, assessment of factor loadings and the proportion of variance explained. The goal of principal component analyses is similar to that of factor analyses, and both approaches often yield the same solutions. However, component loadings are often higher than factor loadings, because the PCA model attempts to account for the entire variance of the correlation matrix, while factor analyses accounts for just the common variance [44, 45]. Therefore, we subsequently conducted confirmatory factor analyses (CFA) to confirm the factor structure. CFA model fit was evaluated using the following criteria [46]: Comparative Fit Index (CFI) ≥ 0.95, Tucker-Lewis Index (TLI) ≥ 0.95, root mean square error of approximation (RMSEA) ≤ 0.06, and standardized root mean square residual (SRMR) ≤ 0.08.

To evaluate internal consistency, Cronbach’s alpha was calculated for each unidimensional (sub)scale. An alpha > 0.7 was considered sufficient. Construct validity was assessed by using Spearman correlations between (sub)scales of the PROMs used in this study. Test-retest reliability of each (sub)scale of PAI-CY was investigated using interclass correlation coefficients (ICCs). A two-way random effect model with absolute agreement was used. Values < 0.5 are indicative of poor reliability, values between 0.5 and 0.75 indicate moderate reliability, values between 0.75 and 0.9 indicate good reliability, and values > 0.90 indicate excellent reliability. Furthermore, the standard error of measurement was calculated using formula SEM = SD*sq root(1-ICC). Table 2 shows the results of the other measurement properties, with sufficient internal consistency and test-retest reliability parameters, and 60% correlations as expected. Cronbach’s alpha for the subscales of PedEyeQ 5–11 ranged from 0.75 to 0.86. Cronbach’s alpha for subscales of the PedEyeQ 12–17 ranged from 0.81 to 0.83. Cronbach’s alpha for the LVP-FVQ II was 0.97.

**Table 2:** Internal consistency, test-retest reliability and correlations between PROM

	PAI-CY (7–12) *N* = 165	PAI-CY (13–17) *N* = 135	
		Factor 1 Physical functioning	Factor 2 Psychosocial functioning
Cronbach’s alpha	0.96	0.96	0.80
Correlation with other PROMs			
LVP-FVQ II	0.67 (expected ≥ 0.6)	0.81 (expected ≥ 0.6)	0.31 (expected 0-3-0.6)
PedEyeQ (5–11)			
Functional vision	-0.16 (expected ≥ 0.6)		
Bothered by eyes/vision	-0.21 (expected < 0.3)		
Social	-0.28 (expected 0.3–0.6)		
Frustration/worry	-0.26 (expected < 0.3)		
PedEyeQ (12–17)			
Functional vision	-	-0.05 (expected ≥ 0.6)	0.24 (expected < 0.3)
Bothered by eyes/vision	-	0.16 (expected < 0.3)	0.11 (expected 0.3–0.6)
Social	-	0.13 (expected < 0.3)	0.04 (expected 0.3–0.6)
Frustration/worry	-	0.04 (expected < 0.3)	0.06 (expected 0.3–0.6)
ICC	0.8 (95% CI 0.9 to 0.3)	1.0 (95% CI 0.9 to 1.0)	0.6 (95% CI 0.4 to 0.8)
SEM	11.3	9.7	2.0

ICC: Intraclass Correlation Coefficient, SEM: Standard Error of Measurement, CI: Confidence Interval

A total of 165 children completed the PAI-CY 7–12 for which subsequent analyses were conducted. Supplementary Table 1 shows the distribution of responses over the response categories and missing scores. The item “How difficult it is for you to read braille” had > 50% missing response and was therefore deleted. Response category 4 “impossible” was found to be used infrequently and was therefore combined with category 3 “very difficult”. The interitem correlation was found to be > 0.7 in 6 item pairs indicating potential redundancy. For the items “How difficult it is for you to brush your teeth” and “How difficult for you to go to the rest room/toilet independently” 90% of children opted response category 1 “not difficult” and hence these items were deleted. Visual inspection of the scree plot (Supplementary Fig. 1), the factor loadings, and the explained variance of the first factor in relation to a second factor (Supplementary Table 1) suggested a one factor model. However, although CFA revealed fit indices approaching satisfactory values for the RMSEA (0.082) and SRMR (0.085), the other fit indices CFI (0.6) and TLI (0.6) were suboptimal.

A total of 135 children completed PAI-CY 13–17 for which subsequent analyses were conducted. Supplementary Table 2 shows the distribution of responses over the response categories and missing scores. The item “How difficult it is for you to handle or deal with menstruation” had > 50% missing response and was therefore deleted. Response category 4 “impossible” was found to be used infrequently hence it was combined with category 3 “very difficult”. The interitem correlation was found to be > 0.7 in 5 item pairs indicating potential redundancy. For the items “How difficult it is for you to participate actively in a conversation (for example during dinner)”, “brush your teeth independently”, “shower/bath independently” and “to do/style your hair” >90% of children chose response category 1 “not difficult” and hence these items were deleted. Visual inspection of scree plot (Supplementary Fig. 2), the factor loadings, and the explained variance (Supplementary Table 2) suggested a two-factor model. The first factor represented ‘physical functioning’ items whereas the second factor represented ‘psychosocial functioning’ items. However, although CFA revealed fit indices approaching satisfactory values for the RMSEA (0.11) and SRMR (0.10), fit indices for the CFI (0.6) and TLI (0.6) were suboptimal. For the LVP-FVQ II and PedEyeQ, the distribution of responses over the response categories was assessed (Supplementary Table 3), as well as the internal consistency.

### Statistical analyses

Data were analyzed using SPSS version 27. Demographic and clinical characteristics were analyzed using descriptive statistics. Total scores were calculated for the PAI-CY 7–12 years whereas subscale scores were calculated for the PAI-CY 13–17 when ≥ 75% of the items were completed. Total scores for the LVP-FVQ II were calculated as well, whereas subscale scores were calculated for the PedEyeQ.

To compare participation and quality of life between children with moderate to severe VI, blindness and children with normal sight (aim1), scores were transformed into 0-100 and compared through independent sample t test and ANOVA. Bonferroni post hoc test was done to all significant variables found in ANOVA. The data distributions were checked using the histogram. Box plots were created to visually present the results. For the PAI-CY, higher scores represent more limitations in activities and participation. Moreover, for the LVP-FVQ II, higher scores represent worse functional vision whereas for PedEyeQ higher scores represent better quality of life. Clinical significance of the differences was investigated using Cohen’s D. Effect sizes 0.2 to 0.49 were considered small, 0.5 to 0.79 were considered moderate, and ≥ 0.8 were considered large [47]. The p value was considered significant if it was < 0.05.The association between severity of VI and participation and quality of life (aim2) was assessed with linear regression analysis after assumptions of normality and linearity were checked. Analyses adjusted for age and sex were performed as well.

### Missing data analysis

All 300 children participated in the first interview. The repeat interview (for test-retest reliability of the PAI-CY) was completed by 60% of the children. The missing data in the repeat interview was caused by the COVID-19 pandemic, which affected data collection.

## Results

A total of 300 children completed the PROMs: 100 children with blindness from integrated schools for the blind, 100 age and sex matched normally sighted peers from the same grade level and class and 100 children with moderate/severe VI recruited from the outpatient department of the Tilganga Institute of Ophthalmology. There were 165 (55%) children in the age group of 7 to 12 years (mean age 9.5, SD 1.7) and 135 (45%) children in the age group of 13 to 17 years (mean age 15.5, SD 1.5). In total, 60% of children were male. The most common cause of blindness and VI was due to retinal diseases followed by whole globe anomalies. Table 3 shows the demographic and clinical characteristic of the participants.

**Table 3:** Demographic and clinical characteristics of participants (*n* = 300)

Characteristics	
**VA category**	N (%)
Normally sighted (VA > 6/12)	100 (33.3)
Moderate to severe VI	100 (33.3)
-Moderate VI (< 6/18 to 6/60)	-90 (90.0)
-Severe VI (< 6/60 to -3/60)	-10 (10.0)
Blind (< 3/60-NLP)	100 (33.3)
Age in years: mean (SD)	12.0 (3.2)
**Gender**	N (%)
-Female	120 (40.0)
-Male	180 (60.0)
**Living situation**	N (%)
Children with blindness	100 (50.0)
-Hostel	91 (91.0)
-Day school	9 (9.0)
Normally sighted children	100 (50.0)
-Hostel	10 (10.0)
-Day scholar	90 (90.0)
**Etiology of VI and blindness**	N (%)
Whole globe	41 (20.5)
Cornea	17 (8.5)
Uvea	21 (10.5)
Lens	25 (12.5)
Retina	71 (35.5)
Optic nerve	15 (7.5)
Other (refractive error, amblyopia, CVI, nystagmus)	29 (14.5)

CVI: Cortical Visual Impairment

**Fig. 1:**
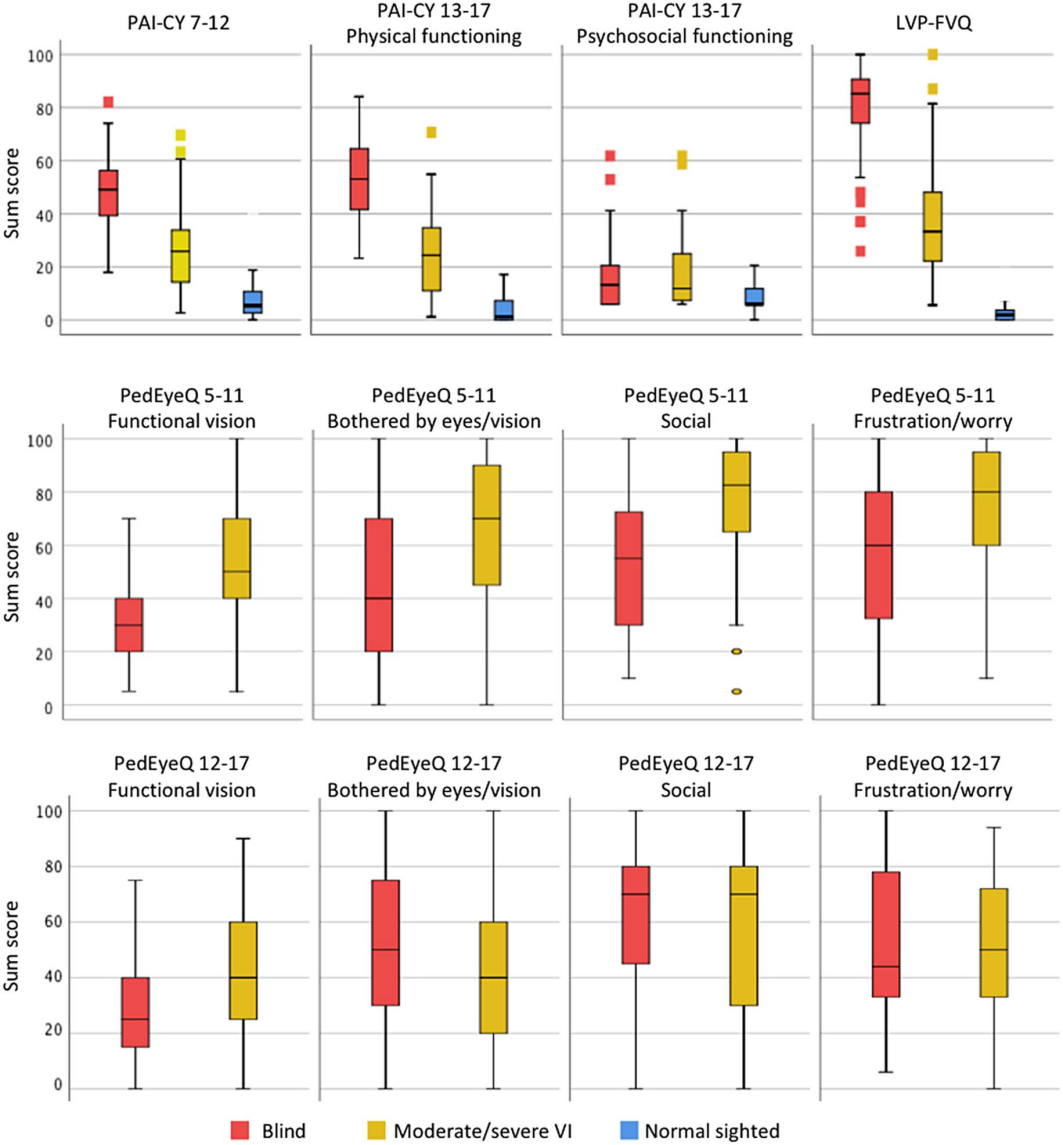
PROM (subscale) scores of children with blindness, moderate/severe visual impairment and normal sight. Boxes represent 1st quartile, median and 3rd quartile values; whiskers represent extreme values

With respect to limitations in activities and participation, blind children scored significantly higher, and thus worse than children with VI, who scored significantly higher than normally sighted children on the PAI-CY 7–12 (Fig. 1; Table 4). The effect sizes for the differences were all large. The same result was found for the physical functioning subscale of the PAI-CY 13–17. For the psychosocial functioning subscale, children with blindness and VI scored significantly higher, and thus worse than children with normal sight, but children with VI scored slightly higher than children with blindness thus having worse participation as represented by this subscale. On the LVP-FVQ II, blind children scored significantly higher and thus worse than children with VI, who scored significantly higher than normally sighted children. With respect to eye-related quality of life, young children (5–11 years) with blindness had significantly lower scores and thus worse scores than children with VI on all four subscales of the PedEyeQ. On the other hand, older children (12–17 years) with blindness only scored significantly lower and thus worse than children with VI on the functional vision subscale. However, in the other three subscales, blind children scored higher than the children with VI, albeit non-significantly.

**Table 4:** Comparison of participation, activities and quality of life scores of children with blindness, moderate/severe VI and normally sighted children

PROM ^a^	Mean Scores (SD)			*P* value	Effect size		
	Normal sighted	Moderate/severe VI	Blind		Normal vs. moderate/ severe VI	Normal vs. blind	Moderate/ severe VI vs. blind
**PAI-CY (7–12)**	*N* = 50	*N* = 69	*N* = 46				
	10.3 (12.4)	29.7 (17.3)	54.1 (15.8)	< 0.001	1.29 (*p* < 0.001)	3.08(*p* < 0.001)	1.47(*p* < 0.001)
**PAI-CY (13–17)**	*N* = 50	*N* = 31	*N* = 54				
- Physical functioning	3.0 (4.6)	20.5 (14.2)	42.9 (12.6)	< 0.001	1.65 (*p* < 0.001)	4.26(*p* < 0.001)	1.66(*p* < 0.001)
- Psychosocial functioning	3.3 (2.7)	6.2 (5.0)	5.8 (4.2)	0.001^b^	0.72 (*p* = 0.004)	0.70(*p* = 0.005)	0.08(*p* = 1.0)
**LVP-FVQ II**	*N* = 100	*N* = 100	*N* = 100				
	1.2 (3.0)	16.8 (9.4)	38.1 (7.4)	< 0.001	2.23 (*p* < 0.001)	6.5(*p* < 0.001)	2.5(*p* < 0.001)
**PedEyeQ (5–11)**	NA	*N* = 58	*N* = 35				
Functional vision		10.5 (4.3)	6.4 (3.5)	< 0.001			1.04
Bothered by eyes/vision		13 (5.8)	8.7 (5.8)	0.001			0.74
Social		15.4 (4.8)	10.7 (5.4)	< 0.001			0.91
Frustration/worry		14.1 (5.3)	11.2 (5.7)	0.008			0.52
**PedEyeQ (12–17)**	NA	*N* = 41	*N* = 65				
Functional vision		8.5 (4.4)	5.4 (3.9)	0.001			0.74
Bothered by eye and vision		8.5 (5.8)	10.3 (5.5)	0.125			0.30
Social		11.5 (5.9)	12.2 (5.0)	0.749			0.12
Frustration/worry		9.1 (4.8)	9.4 (4.7)	0.302			0.06

^a^ PAI-CY and LVP-FVQ II: higher scores represent worse functioning/participation; PedEyeQ: higher scores represent better quality of life; ^b^ No significant difference between moderate/severe VI and blind

The uncorrected model showed that both moderate/severe VI and blindness were significantly associated with worse scores on the PAI-CY 7–12, the two subscales of the PAI-CY 13–17, and the LVP-FVQ II (Table 5). After adjusting for age and sex, these associations remained. Blindness was significantly associated with worse scores on all four subscales of the PedEyeQ 5–11 compared to moderate/severe VI. Similar results were found after adjusting for age and sex. Blindness was also significantly associated with worse scores on the functional vision subscale of the PedEyeQ 12–17, both in the uncorrected and corrected model. In contrast, blindness was associated with better scores on other three subscales of the.

PedEyeQ 12–17 compared to moderate/severe VI, but these associations were not significant in the crude model, nor after correcting for age and sex.

**Table 5:** Association between severity of vision loss and scores on different proms and their subscales

PROMs^a^	Crude model β (95%CI)	*P* value	Adjusted model^b^ β (95%CI)	*P* value
**PAI-CY (7–12)** ^c^				
Moderate/severe VI	19.3 (13.6 to 25.1)	< 0.001	18.3 (12.5 to 24.0)	< 0.001
Blind	43.8 (37.5 to 50.1)	< 0.001	43.8 (37.6 to 50.0)	< 0.001
**PAI-CY (13–17)** ^**c**^				
-Physical functioning				
Moderate/severe VI	13.7 (7.4 to 20.1)	< 0.001	12.6 (6.1 to 19.0)	< 0.001
Blind	31.0 (26.0 to 36.0)	< 0.001	31.1 (26.1 to 36.1)	< 0.001
-Psychosocial functioning				
Moderate/severe VI	3.0 (1.1 to 4.7)	0.002	3.0 (1.2 to 4.1)	0.001
Blind	2.5 (1.0 to 4.1)	0.001	3.0 (1.4 to 4.4)	< 0.001
**LVP-FVQ** ^**c**^				
Moderate/severe VI	18.2 (16.0 to 21.0)	< 0.001	18.0 (15.7 to 20.2)	< 0.001
Blind	41.7 (39.5 to 43.9)	< 0.001	41.9 (39.7 to 44.1)	< 0.001
**PedEyeQ (5–11)** ^d^				
-Functional vision				
Blind	-4.1 (-5.8 to -2.4)	< 0.001	-4.2 (-6.0 to -2.5)	< 0.001
-Bothered about eyes/vision				
Blind	-4.3 (-6.8 to -1.8)	< 0.001	-4.6 (-7.1 to -2.0)	0.001
-Social				
Blind	-4.7 (− 6.9 to − 2.6)	< 0.001	-4.6 (− 6.8 to − 2.4)	< 0.001
-Frustration/worry				
Blind	-3.3 (− 5.6 to − 0.9)	0.006	-3.0 (-5.3 to -5.9)	0.015
**PedEyeQ (12–17)** ^**d**^				
-Functional vision				
Blind	-3.0 (-4.7 to -1.4)	< 0.001	-3.0 (-4.7 to -1.4)	< 0.001
-Bothered about eyes/vision				
Blind	1.8 (-0.5 to 4.0)	0.118	1.9 (-0.4 to 4.1)	0.101
-Social				
Blind	0.7 (-1.4 to 2.9)	0.490	0.8 (-1.4 to 3.0)	0.466
-Frustration/worry				
Blind	0.3 (-1.6 to 2.1)	0.751	0.3 (-1.7 to 2.2)	0.797

^a^ PAI-CY and LVP-FVQ: higher scores represent worse functioning/participation; PedEyeQ: higher scores represent better quality of life; ^b^ Corrected for age and sex; ^c^ Normal sighted served as a reference group; ^d^ Moderate/severe VI served as a reference group

## Discussion

This study reports on potential limitations in activities and participation, functional vision and eye-related quality of life of Nepalese children aged 7 to 17 years with VI as assessed with respectively the PAI-CY, LVP-FVQ II and PedEyeQ. We compared children with blindness, moderate to severe VI and normal sight. Furthermore, this study provides insight into the association between vision loss and these health outcomes.

Children with VI and blindness experienced more limitations in activities and participation than children with normal sight in both age groups. This is consistent with multiple previous studies. For example, Elsman et al. found that children with VI living in a high-income country scored worse on participation than a normally sighted reference population [22]. A study by Khadka et al. showed that, although lifestyles of children and adolescents with VI are similar to that of normally sighted peers, they experience participation restrictions in leisure time activities, sports and social interaction [17]. Similarly, a study conducted by Engel et al. in Israel found participation limitations in children with VI compared to normally sighted children expressed as a lower number of activities, lower participation intensity, more activities performed at home and with someone else. However, their sample size was very small [48]. Similar results were also found in a small study conducted in Iran that used parent-reported information and found that there were meaningful differences between children with VI and normal sight in overall participation, nutrition, communication, participation at home, mobility, responsibility, interpersonal relationships, education, and recreation [49]. In contrast to the above-mentioned studies, we did not evaluate differences at the domain level but compared overall participation and activity scores, as the PAI-CY uses the domains only descriptively. Our analyses for structural validity revealed that the PAI-CY 7–12 comprises a unidimensional scale whereas the PAI-CY 13–17 comprises two subscales.

After further analyzing the association between severity of VI with limitations in activities and participation, we found that children 7 to 12 years with moderate/severe VI experienced fewer limitations than children with blindness. This was also consistent with the findings of Elsman et al. who found that more severe VI is associated with more limitations in participation. The same result was found for the physical functioning subscale in children 13 to 17 years. However, for the psychosocial functioning subscale, only small differences in scores between children with blindness, moderate/severe VI and normal sight were observed, and children with blindness scored slightly better on activities and participation than children with moderate/severe VI. The reason that children with blindness had similar psychosocial outcomes as children with VI might be found in the integrated system of education in Nepal. Several studies have highlighted the benefits of an integrated system of education for children with VI or blindness [50–54]. However, Erwin et al. did not find any difference between integrated or exclusive schools, but because of their small sample size, they advised further research [55]. Another explanation could be that 90% of children with blindness in our study lived in hostels. This might help children to bond with each other and improve their psychosocial functioning. On the other hand, despite having a better functional vision and physical functioning, children with VI in the older age group scored worse on psychosocial functioning than children with blindness, although this was not statistically significant. This might indicate that children with VI lack proper low vision support services for example information on the availability of low vision devices and proper training in using these devices. Because of this, they may face difficulty in getting along with mainstream education. Moreover, these children have to deal with parental expectations as well as expectations from themselves to compete with their normally sighted peers, which might lead to mental health issues [56–58].

We used LVP-FVQ II to assess functional vision in children with VI and it showed that these children had worse functional vision compared to normally sighted peers. There was an association between severity of VI and worsening scores on the LVP-FVQ II. This finding is similar to the study by Chadha et al. and Catherine et al. [20, 37].

We found that younger children with blindness had worse quality of life as measured with the PedEyeQ than children with moderate/severe VI for all subscales. This is in line with the findings of a study by Leske et al. [59], but in contrast to the findings of Elsman et al., they found mixed results on different quality of life subscales although these were measured with a different PROM. Furthermore, they did not find any clear trend for worse quality of life with more severe VI. In our current study we did find such a trend for younger children, but not for older children, except for the functional vision subscale. These results again might indicate a lack of adequate low vision support services in Nepal for children with moderate to severe VI and blindness. Furthermore, younger children with moderate to severe VI showed better quality of life than the older children on all subscales except for functional vision. This is in contrast to the findings by Elsman et al. who found worse quality of life in younger children related to psychosocial wellbeing, autonomy, parental relationship and school environment.

There are several limitations to be mentioned. First, the PedEyeQ was not administered in children with normal sight. The content of this questionnaire is focused on eye diseases and conditions and as such is irrelevant for children with normal sight. Second, we were not able to further divide the children in moderate or severe VI because the number of children in the severe VI group was very low compared to the moderate VI group. Therefore, we could not compare scores for these sub categories of vision loss which would potentially have given a more detailed insight into the association between severity of VI with participation and quality of life. Third, our study was conducted in schools selected through purposive sampling and in the hospital selected through consecutive sampling, which might have introduced selection bias. Although most children with VI come from different corners of Nepal and reside in hostels in the Kathmandu Valley, there might be some children in the community who do not attend schools and may be expected to have worse participation and activity profiles than the school going children. Fourth, the repeat interview could be completed by only 60% of children because of the COVID-19 pandemic which affected data collection. Lastly, although children with VI had a diverse etiology of diseases we did not analyze them separately because of the small sample size in each subgroup of diseases. Despite these limitations, to our knowledge, this is the largest comparative study on participation and quality of life of children with VI and normal sight by using children’s self-report. We did not administer proxy-reports by parents as this could introduce bias as parents might compare their VI children with normally sighted siblings or peers. A second strength is the extensive evaluation of the measurement properties of the PAI-CY after we culturally adapted the questionnaire for the purpose of this study. The cultural adaptation of translated questionnaires was carried out by a panel of experts and the questionnaires were pretested to evaluate comprehensibility and relevance among children with visual impairment and blindness in Nepal [43].

We did not evaluate the measurement properties of LVP-FVQ II and PedEyeQ but checked some basic properties as they were only translated and have been extensively evaluated previously in countries with a similar cultural background [60, 61].

## Conclusion

In conclusion, this study shows that activity and participation as well as functional vision are affected in children with VI compared to normally sighted peers. Younger children with blindness experienced a worse quality of life compared to children with VI although results for older children showed a mixed pattern. This study helps to understand the limitations in activity and participation in children with VI experience and how their quality of life is affected, which is valuable for children, parents, care givers, teachers and health care professionals working with these children. However, we were unable to include children who do not attend school and may have worse participation and quality of life outcomes than school going children. Appropriate low vision rehabilitation interventions and devices might be needed, and further study might help to explore the availability of low vision rehabilitation programs in Nepal and barriers or facilitators for their implementation.

## Electronic supplementary material

Below is the link to the electronic supplementary material.

Supplementary Material 1

Supplementary Material 2

Supplementary Material 3

## Data Availability

The data sets used and or analyzed during the current study are available from the corresponding author on reasonable request.

## Abbreviations

VI: Visual Impairment
PAI-CY: Participation and Activity Inventory - Children and Youth
LVP-FVQ: L.V. Prasad Functional Vision Questionnaires
PedEyeQ: Pediatric Eye Questionnaires
PROMs: Patient Reported Outcome Measures
NPVIS: Nepal Pediatric Visual Impairment Study
BCVA: Best corrected Visual Acuity
CFA: Confirmatory Factor Analysis
RMSEA: Root Mean Square Error of Approximation
SRMR: Standardized Post Mean Square Residual
ICC: Intraclass correlation coefficient
SEM: Standard Error of Mean
CFI: Comparative fit index
TLI: Tucker Lewis Index
