# Supplementary material for: Participation and quality of life of Nepalese children with visual impairment in comparison with normally sighted peers: a cross sectional comparative study
Source: J Patient Rep Outcomes. 2025 Jun 5;9:64. doi: 10.1186/s41687-025-00893-2 (PMC12141697; doi:10.1186/s41687-025-00893-2)
Supplement: Supplementary file 1 — Supplementary Material 1 [file 41687_2025_893_MOESM1_ESM.docx]

STROBE Statement—checklist of items that should be included in reports of observational studies

|  | Item No. | Recommendation | Page  No. | Relevant text from manuscript |  |  |
| --- | --- | --- | --- | --- | --- | --- |
| **Title and abstract** | 1 | (*a*) Indicate the study’s design with a commonly used term in the title or the abstract | 1 | Participation and quality of life of Nepalese children with visual impairment in comparison with normally sighted peers: A cross sectional comparative study |  |  |
|  |  | (*b*) Provide in the abstract an informative and balanced summary of what was done and what was found | 2 | Background  Poor vision compromises quality of life and participation in different daily life activities of children such as, sports, leisure time, interactive play and social interaction. The purpose of this study is to investigate participation and quality of life of children with visual impairment (VI) and blindness compared with normally sighted peers.  Methodology  Children aged 7-17 years with blindness (n=100), moderate to severe VI (n=100) and normal sight (n=100) completed Nepalese versions of the Participation and activity inventory children and youth (PAI-CY 7-12 and 13-17), L. V. Prasad functional vision questionnaires (LVP-FVQ II ) and Pediatric Eye Questionnaires (PedEyeQ 5-11 and 12-17). The measurement properties of PAI -CY was studied. All (sub) scores were compared between three groups. Associations between the severity of VI and outcomes were assessed with age and sex adjusted linear regression analyses.  Results  Children with blindness scored worse than children with VI, who scored worse than normally sighted children on the PAI-CY 7-12, the physical functioning subscale of the PAI-CY 13-17 and LVP-FVQ II(p=<0.001). However, for the psychosocial functioning subscale of PAI-CY 13-17, children with blindness scored better than children with VI(p=<0.01). On the PedEyeQ, young children (5-11) with blindness on all subscales, and older children (12-17 years) with blindness on the functional vision subscale scored worse than children with VI(p=<0.01). In other subscales, they scored higher than children with VI but not statistically significant. Regression models showed that both moderate/severe VI and blindness were significantly associated with worse PAI-CY, LVP-FVQ II, PedEyeQ 5-11 and PedEyeQ 12-17 functional vision subscale scores(p=<0.01).  Conclusion  Younger children with blindness showed worse participation and quality of life compared to children with VI, whereas results for older children showed a mixed pattern with children with blindness showing better participation in psychosocial domain. Appropriate low vision rehabilitation interventions are needed for children with VI and blindness to increase their participation and quality of life to the level of their normally sighted peers as far as possible. Further studies recommended to include children in the community who do not attend school and may have worse participation and activity profiles than the school going children |  |  |
| Introduction | | |  | |  |  |
| Background/rationale | 2 | Explain the scientific background and rationale for the investigation being reported | 4 | Measuring quality of life and participation in children and adolescents with VI can be considered important throughout the course of their development. Children with VI are the best judges of their own activity limitations, participation, and quality of life. In recent years patient-reported outcome measures (PROMs) have been developed specifically for children with VI. Most PROMs focus on vision-related quality of life or functional vision which refers to how well an individual performs while interacting with the visual environment ^24-29^ For example, the LV Prasad Functional Vision Questionnaire (LVP-FVQ) was the designed to assess functional vision in children with VI in developing countries ^30-31^, whereas the Pediatric Eye Questionnaire (PedEyeQ) was developed to measure functional vision and eye-related quality of life in children with any eye condition.^32^ Moreover, the Participation and Activity Inventory for Children and Youth (PAI-CY) consists of a series of PROMs to measure limitations in activities and participation in children with VI in different age groups from 0-17 years. The PAI-CY was originally developed in a Dutch paediatric population. ^33-38^  Although a handful of studies is available from neighbouring countries like China, the Philippines and India ^39,23,40^, little is known about participation and quality of life of children with VI in Nepal. Therefore, we recently translated, culturally adapted and pilot tested the PAI-CY 7-12 and 13-17 in the Nepali language ^41^ |  |  |
| Objectives | 3 | State specific objectives, including any prespecified hypotheses | 4,5 | The aim of the current study was to 1) investigate limitations in activities and participation, and quality of life of children with VI and to compare these outcomes with normally sighted peers and 2) to evaluate the association between the severity of vision loss and these important health outcomes. |  |  |
| Methods | | |  | |  |  |
| Study design | 4 | Present key elements of study design early in the paper |  | This cross-sectional study is a part of the Nepal Pediatric Vision Impairment Study (NPVIS) which aims to study activity, participation, sleep and quality of life of children with VI compared to normally sighted peers. |  |  |
| Setting | 5 | Describe the setting, locations, and relevant dates, including periods of recruitment, exposure, follow-up, and data collection |  | The study was carried out in five integrated schools for the blind in and around the Kathmandu valley and the Tilganga Institute of Ophthalmology over a period of 14 months, from April 2021 to June 2022. |  |  |
| Participants | 6 | (*a*) *Cohort study*—Give the eligibility criteria, and the sources and methods of selection of participants. Describe methods of follow-up  *Case-control study*—Give the eligibility criteria, and the sources and methods of case ascertainment and control selection. Give the rationale for the choice of cases and controls  *Cross-sectional study*—Give the eligibility criteria, and the sources and methods of selection of participants |  | Through purposive sampling, children with blindness (N=100) and normally sighted children (N=100) were recruited from the integrated schools for the blind. Children with moderate and severe VI (N=100) were recruited through consecutive sampling from the outpatient department of the Tilganga Institute of Ophthalmology in Kathmandu.Participants included children aged between 7 and 17 years. Children with blindness (best corrected visual acuity (BCVA) <3/60 in the better eye), were age- and sex-matched to normally sighted peers (BCVA ≥ 6/12) from the same school and grade level. Participants with moderate (BCVA <6/12 to 6/60) and severe (BCVA <6/60 to 3/60) VI were those who visited the outpatient department of the Tilganga Institute of Ophthalmology in Kathmandu during the study period. Children with developmental delay, cognitive impairment or other disabilities apart from VI were excluded from the study. |  |  |
|  |  | (*b*) *Cohort study*—For matched studies, give matching criteria and number of exposed and unexposed  *Case-control study*—For matched studies, give matching criteria and the number of controls per case |  | Children with blindness (best corrected visual acuity (BCVA) <3/60 in the better eye), were age- and sex-matched to normally sighted peers (BCVA ≥ 6/12) from the same school and grade level. |  |  |
| Variables | 7 | Clearly define all outcomes, exposures, predictors, potential confounders, and effect modifiers. Give diagnostic criteria, if applicable |  | Demographic information including age, sex and living condition (hostel vs. day scholar) was collected. Each child underwent a vision assessment which consisted of visual acuity examination. Children with VI additionally underwent anterior and posterior segment evaluation. All children completed PROMs through face-to-face interviews by a community health worker, which were audio recorded for later verification. A repeat interview to assess test-retest reliability was carried out 2 weeks following the initial interview. PROMs included the Nepalese versions of the PAI-CY 7-12, or 13-17, depending on the age of the child, and the Nepalese version of the LVP-FVQ II and PedEyeQ 5-11 or 12-17. Table 1 details which PROMs were completed by which subgroups.  Table 1: PROMs completed by different subgroups  The Nepalese version of the PAI-CY 7-12 consists of 61 items which cover nine domains (for descriptive purposes only): play, social contact, mobility, leisure time, communication, school, self-reliance, acceptance/self-consciousness and finance. The Nepalese version of PAI-CY 13-17 consists of 68 items which cover eight domains: leisure time, mobility, social contact, communication, school, self-reliance, acceptance/self-consciousness and finance. Responses to the items on both versions are provided on a four-point Likert scale: (1) not difficult; (2) slightly difficult; (3) very difficult; and (4) impossible. The response option ‘not applicable’ was treated as missing value.  The LVP-FVQ II contains 23 items which are answered on a four-point Likert type scale: (1) no difficulty; (2) some difficulty; and (3) a lot of difficulty. The questions are designed to cover four domains: distance vision, near vision, color vision and visual field, but a total score is calculated. It was developed for children 8-17 years, however, we used it in children between 7-17 years.  The child versions of the PedEyeQ 5-11 and 12-17 were used, depending on the age of the child. Both versions consist of four domains with 10 items in each domain: functional vision, bothered by eyes/vision, social and frustration/worry. Responses to the items are provided on a three-point Likert scale: (2) never; (1) sometimes; and (0) all of the time. |  |  |
| Data sources/ measurement | 8* | For each variable of interest, give sources of data and details of methods of assessment (measurement). Describe comparability of assessment methods if there is more than one group |  | Total scores were calculated for the PAI-CY 7-12 years whereas subscale scores were calculated for the PAI-CY 13-17 when ≥75% of the items were completed. Total scores for the LVP-FVQ II were calculated as well, whereas subscale scores were calculated for the PedEyeQ.  To compare participation and quality of life between children with moderate to severe VI, blindness and children with normal sight, scores were transformed into 0-100. |  |  |
| Bias | 9 | Describe any efforts to address potential sources of bias |  | N/A |  |  |
| Study size | 10 | Explain how the study size was arrived at |  | Through purposive sampling, children with blindness (n=100) and normally sighted children (n=100) were recruited from the integrated schools for the blind. Children with moderate and severe VI (n=100) were recruited through consecutive sampling from the outpatient department of the Tilganga Institute of Ophthalmology in Kathmandu. |  |  |
| Quantitative variables | 11 | Explain how quantitative variables were handled in the analyses. If applicable, describe which groupings were chosen and why |  | To compare participation and quality of life between children with moderate to severe VI, blindness and children with normal sight, scores were transformed into 0-100 | |  |
| Statistical methods | 12 | (*a*) Describe all statistical methods, including those used to control for confounding |  | Previously we culturally adapted the PAI-CY for use in Nepal [38] and therefore some measurement properties were studied. First, item analyses for the two age versions of the PAI-CY were performed to investigate floor and ceiling effects at the item level and distribution over the response categories. Items with >50% missing responses were deleted. Items with an inter-item correlation >0.7 were flagged for potential redundancy. Next, structural validity was first investigated through principal component analysis (PCA). The number of factors was examined through a combination of visually inspecting the scree plots, assessment of factor loadings and the proportion of variance explained. The goal of principal component analyses is similar to that of factor analyses, and both approaches often yield the same solutions. However, component loadings are often higher than factor loadings, because the PCA model attempts to account for the entire variance of the correlation matrix, while factor analyses accounts for just the common variance. ^42-43^Therefore, we subsequently conducted confirmatory factor analyses (CFA) to confirm the factor structure. CFA model fit was evaluated using the following criteria. ^44^: Comparative Fit Index (CFI) ≥0.95, Tucker-Lewis Index (TLI) ≥0.95, root mean square error of approximation (RMSEA) ≤0.06, and standardized root mean square residual (SRMR) ≤0.08.  To evaluate internal consistency, Cronbach’s alpha was calculated for each unidimensional (sub)scale. An alpha >0.7 was considered sufficient. Construct validity was assessed by using Spearman correlations between (sub)scales of the PROMs used in this study. Test-retest reliability of each (sub)scale of PAI-CY was investigated using interclass correlation coefficients (ICCs). A two-way random effect model with absolute agreement was used. Values <0.5 are indicative of poor reliability, values between 0.5-0.75 indicate moderate reliability, values between 0.75-0.9 indicate good reliability, and values >0.90 indicate excellent reliability. Furthermore, the standard error of measurement was calculated using formula SEM=SD*sq root(1-ICC). […]  Data was analyzed using SPSS version 27. Demographic and clinical characteristics were analyzed using descriptive statistics. Total scores were calculated for the PAI-CY 7-12 years whereas subscale scores were calculated for the PAI-CY 13-17 when ≥75% of the items were completed. Total scores for the LVP-FVQ II were calculated as well, whereas subscale scores were calculated for the PedEyeQ.  To compare participation and quality of life between children with moderate to severe VI, blindness and children with normal sight, scores were transformed into 0-100 and compared through independent samples t-tests and ANOVAs. For the PAI-CY, higher scores represent more limitations in activities and participation. Moreover, for the LVP-FVQ II, higher scores represent worse functional vision whereas for PedEyeQ higher scores represent better quality of life. Clinical significance of the differences was investigated using Cohen's D. Effect sizes 0.2 to 0.49 were considered small, 0.5 to 0.79 were considered moderate, and ≥0.8 were considered large.^45^ The p value was considered significant if it was <0.05The association between severity of VI and participation and quality of life was assessed with linear regression analysis after assumptions were checked. Analyses adjusted for age and sex were performed as well. | |  |
|  |  | (*b*) Describe any methods used to examine subgroups and interactions |  | N/A | |  |
|  |  | (*c*) Explain how missing data were addressed |  | Items with >50% missing responses were deleted. Total scores were calculated for the PAI-CY 7-12 years whereas subscale scores were calculated for the PAI-CY 13-17 when ≥75% of the items were completed. All 300 children participated in the first interview. The repeat interview (for test-retest reliability of the PAI-CY) was completed by 60% of the children. | |  |
|  |  | (*d*) *Cohort study*—If applicable, explain how loss to follow-up was addressed  *Case-control study*—If applicable, explain how matching of cases and controls was addressed  *Cross-sectional study*—If applicable, describe analytical methods taking account of sampling strategy |  | N/A | |  |
|  |  | (*e*) Describe any sensitivity analyses |  | N/A | |  |
| Results | | | | |  |  |
| Participants | 13* | (a) Report numbers of individuals at each stage of study—eg numbers potentially eligible, examined for eligibility, confirmed eligible, included in the study, completing follow-up, and analysed |  | A total of 300 children completed the PROMs: 100 children with blindness from integrated schools for the blind, 100 age and sex matched normally sighted peers from the same grade level and class and 100 children with moderate/severe VI recruited from the outpatient department of the Tilganga Institute of Ophthalmology. There were 165 (55%) children in the age group of 7 to 12 years (mean age 9.5, SD 1.7) and 135 (45%) children in the age group of 13 to 17 years (mean age 15.5, SD 1.5). | |  |
|  |  | (b) Give reasons for non-participation at each stage |  | While doing repeat interview only 60 % of children were present. This is because of the COVID 19 Pandemic which affected data collection. | |  |
|  |  | (c) Consider use of a flow diagram |  | N/A | |  |
| Descriptive data | 14* | (a) Give characteristics of study participants (eg demographic, clinical, social) and information on exposures and potential confounders |  | In total, 60% of children were male. The most common cause of blindness and VI was due to retinal diseases followed by whole globe anomalies. **Table 3** shows the demographic and clinical characteristic of the participants.  Table 3: Demographic and clinical characteristics of participants (n=300) | |  |
|  |  | (b) Indicate number of participants with missing data for each variable of interest |  | Supplementary table 1 and 2 | |  |
|  |  | (c) *Cohort study*—Summarise follow-up time (eg, average and total amount) |  | N/A | |  |
| Outcome data | 15* | *Cohort study*—Report numbers of outcome events or summary measures over time |  | N/A | |  |
|  |  | *Case-control study—*Report numbers in each exposure category, or summary measures of exposure |  | N/A | |  |
|  |  | *Cross-sectional study—*Report numbers of outcome events or summary measures |  | With respect to limitations in activities and participation, blind children scored significantly higher, and thus worse than children with VI, who scored significantly higher than normally sighted children on the PAI-CY 7-12 (Figure 1, Table 4). The effect sizes for the differences were all large. The same result was found for the physical functioning subscale of the PAI-CY 13-17. For the psychosocial functioning subscale, children with blindness and VI scored significantly higher, and thus worse, than children with normal sight, but children with VI scored slightly higher than children with blindness thus having worse participation as represented by this subscale. On the LVP-FVQ II, blind children scored significantly higher, and thus worse than children with VI, who scored significantly higher than normally sighted children. With respect to eye-related quality of life, young children (5-11 years) with blindness had significantly lower scores and thus worse scores than children with VI on all four subscales of the PedEyeQ. On the other hand, older children (12-17 years) with blindness only scored significantly lower and thus worse than children with VI on the functional vision subscale. However, in the other three subscales, blind children scored higher than the children with VI, albeit non-significantly.  Fig: 1 PROM (subscale) scores of children with blindness, moderate/severe visual impairment and normal sight. Boxes represent 1^st^ quartile, median and 3^rd^ quartile values; whiskers represent extreme values.  Table 4: Comparison of participation, activities and quality of life scores of children with blindness, moderate/severe VI and normally sighted children | |  |
| Main results | 16 | (*a*) Give unadjusted estimates and, if applicable, confounder-adjusted estimates and their precision (eg, 95% confidence interval). Make clear which confounders were adjusted for and why they were included |  | The uncorrected model showed that both moderate/severe VI and blindness were significantly associated with worse scores on the PAI-CY 7-12, the two subscales of the PAI-CY 13-17, and the LVP-FVQ II (Table 5). After adjusting for age and sex, these associations remained. Blindness was significantly associated with worse scores on all four subscales of the PedEyeQ 5-11 compared to moderate/severe VI. Similar results were found after adjusting for age and sex. Blindness was also significantly associated with worse scores on the functional vision subscale of the PedEyeQ 12-17, both in the uncorrected and corrected model. In contrast, blindness was associated with better scores on other three subscales of the PedEyeQ 12-17 compared to moderate/severe VI, but these associations were not significant in the crude model, nor after correcting for age and sex. | |  |
|  |  | (*b*) Report category boundaries when continuous variables were categorized |  | N/A | |  |
|  |  | (*c*) If relevant, consider translating estimates of relative risk into absolute risk for a meaningful time period |  | N/A | |  |
| Other analyses | 17 | Report other analyses done—eg analyses of subgroups and interactions, and sensitivity analyses |  | N/A | | |
| Discussion | | | | | | |
| Key results | 18 | Summarise key results with reference to study objectives |  | This study reports on potential limitations in activities and participation, functional vision and eye-related quality of life of children aged 7 to 17 years with VI as assessed with respectively the PAI-CY, LVP-FVQ II and PedEyeQ. We compared children with blindness, moderate to severe VI and normal sight. Furthermore, this study provides insight into the association between vision loss and these health outcomes.  Children with VI and blindness experienced more limitations in activities and participation than children with normal sight in both age groups. This is consistent with multiple previous studies. For example, Elsman et al. found that children with VI living in a high-income country scored worse on participation than a normally sighted reference population.^22^ A study by Khadka et al. showed that, although lifestyles of children and adolescents with VI are similar to that of normally sighted peers, they experience participation restrictions in leisure time activities, sports and social interaction.^17^ Similarly, a study conducted be Engel et al. in Israel found participation limitations in children with VI compared to normally sighted children expressed as a lower number of activities, lower participation intensity, more activities performed at home and with someone else. However, their sample size was very small. ^46^ Similar results were also found in a small study conducted in Iran that used parent-reported information and found that there were meaningful differences between children with VI and normal sight in overall participation, nutrition, communication, participation at home, mobility, responsibility, interpersonal relationships, education, and recreation. ^47^In contrast to the above-mentioned studies, we did not evaluate differences at the domain level but compared overall participation and activity scores, as the PAI-CY uses the domains only descriptively. Our analyses for structural validity revealed that the PAI-CY 7-12 comprises a unidimensional scale whereas the PAI-CY 13-17 comprises two subscales.  After further analysing the association between severity of VI with limitations in activities and participation, we found that children 7 to 12 years with moderate/severe VI experienced less limitations than children with blindness. This was also consistent with the findings of Elsman et al. who found that more severe VI is associated with more limitations in participation. The same result was found for the physical functioning subscale in children 13 to 17 years. However, for the psychosocial functioning subscale, only small differences in scores between children with blindness, moderate/severe VI and normal sight were observed, and children with blindness scored slightly better on activities and participation than children with moderate/severe VI. The reason that children with blindness had similar psychosocial outcomes as children with VI might be found in the integrated system of education in Nepal. Several studies have highlighted the benefits of an integrated system of education for children with VI or blindness.^48-52^ However, Erwin et al. did not find any difference between integrated or exclusive schools but because of their small sample size, they advised further research. ^53^Another explanation could be that 90% of children with blindness in our study lived in hostels. This might help children to bond with each other and improve their psychosocial functioning. On the other hand, despite having a better functional vision and physical functioning, children with VI in the older age group scored worse on psychosocial functioning than children with blindness, although this was not statistically significant. This might indicate that children with VI lack proper low vision support services for example information on the availability of low vision devices and proper training in using these devices. Because of this, they may face difficulty in getting along with mainstream education. Moreover, these children have to deal with parental expectations as well as expectations from themselves to compete with their normally sighted peers, which might lead to mental health issues. ^54-56^  We used LVP-FVQ II to assess functional vision in children with VI and it showed that these children had worse functional vision compared to normally sighted peers. There was an association between severity of VI and worsening scores on the LVP-FVQ II. This finding is similar to the study by Chadha et al. and Catherine et al.^20,37^  Younger children with blindness had worse quality of life as measured with the PedEyeQ than children with moderate/severe VI for all subscales. This is in line with the findings of a study by Leske et al ^57^, but in contrast to the findings of Elsman et al, they found mixed results on different quality of life subscales although these were measured with a different PROM. Furthermore, they did not find any clear trend for worse quality of life with more severe VI. In our current study we did find such a trend for younger children, but not for older children, except for the functional vision subscale. These results again might indicate a lack of adequate low vision support services in Nepal for children with moderate to severe VI and blindness. Furthermore, younger children with moderate to severe VI showed better quality of life than the older children on all subscales except for functional vision. This is in contrast to the findings by Elsman et al. who found worse quality of life in younger children related to psychosocial wellbeing, autonomy, parental relationship and school environment. | | |
| Limitations | 19 | Discuss limitations of the study, taking into account sources of potential bias or imprecision. Discuss both direction and magnitude of any potential bias |  | There are several limitations to be mentioned. First, the PedEyeQ was not administered in children with normal sight. The content of this questionnaire is focused on eye diseases and conditions and as such is irrelevant for children with normal sight. Second, we were not able to further divide the children in moderate or severe VI because the number of children in the severe VI group was very low compared to the moderate VI group. Therefore, we could not compare scores for these sub categories of vision loss which would potentially have given a more detailed insight into the association between severity of VI with participation and quality of life. Third, our study was conducted in schools and in the hospital, which might have introduced selection bias. Although most children with VI come from different corners of Nepal and reside in hostels in the Kathmandu Valley, there might be some children in the community who do not attend schools and may be expected to have worse participation and activity profiles than the school going children. The repeat interview could be completed by only 60 % of children because of the COVID-19 pandemic which affected data collection. Lastly, although children with VI had a diverse etiology of diseases we did not analyze them separately because of the small sample size in each subgroup of diseases. Despite these limitations, to our knowledge, this is the largest comparative study on participation and quality of life of children with VI and normal sight by using children’s self-report. We did not administer proxy-reports by parents as this could introduce bias as parents might compare their VI children with normally sighted siblings or peers. A second strength is the extensive evaluation of the measurement properties of the PAI-CY after we culturally adapted the questionnaire for the purpose of this study. We did not evaluate the measurement properties of LVP-FVQ II and PedEyeQ but checked some basic properties as they were only translated and have been extensively evaluated previously in countries with a similar cultural background ^58,59^ | | |
| Interpretation | 20 | Give a cautious overall interpretation of results considering objectives, limitations, multiplicity of analyses, results from similar studies, and other relevant evidence |  | In conclusion, this study shows that activity and participation as well as functional vision are affected in children with VI compared to normally sighted peers. Younger children with blindness experienced a worse quality of life compared to children with VI although results for older children showed a mixed pattern. | | |
| Generalisability | 21 | Discuss the generalisability (external validity) of the study results |  | This study helps to understand the limitations in activity and participation in children with VI experience and how their quality of life is affected, which is valuable for children, parents, care givers, teachers and health care professionals working with these children. Appropriate low vision rehabilitation interventions and devices might be needed for these children. Further study might help to explore the availability of low vision rehabilitation programs in Nepal and barriers or facilitators for their implementation. | | |
| Other information | |  | | | | |
| Funding | 22 | Give the source of funding and the role of the funders for the present study and, if applicable, for the original study on which the present article is based |  | The authors declare that no funds, grants, or other support were received during the preparation of this manuscript. | | |

*Give information separately for cases and controls in case-control studies and, if applicable, for exposed and unexposed groups in cohort and cross-sectional studies.

**Note:** An Explanation and Elaboration article discusses each checklist item and gives methodological background and published examples of transparent reporting. The STROBE checklist is best used in conjunction with this article (freely available on the Web sites of PLoS Medicine at http://www.plosmedicine.org/, Annals of Internal Medicine at http://www.annals.org/, and Epidemiology at http://www.epidem.com/). Information on the STROBE Initiative is available at www.strobe-statement.org.
