# Supplementary figures and images for: Participation and quality of life of Nepalese children with visual impairment in comparison with normally sighted peers: a cross sectional comparative study
Source: J Patient Rep Outcomes. 2025 Jun 5;9:64. doi: 10.1186/s41687-025-00893-2 (PMC12141697; doi:10.1186/s41687-025-00893-2)

**Supplementary figure 1:** Scree plot of the PAI-CY 7-12


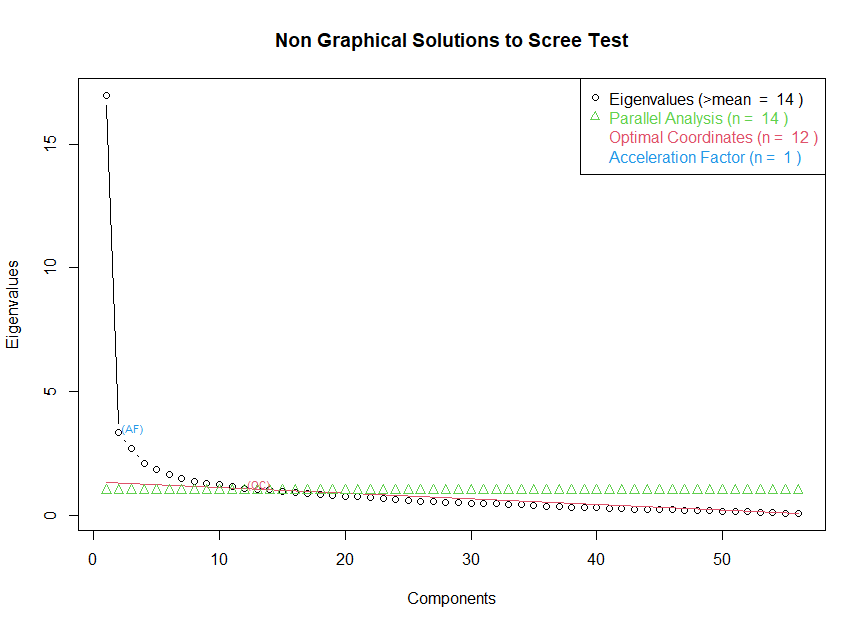


**Supplementary figure 2:** Scree plot of the PAI-CY 13-17


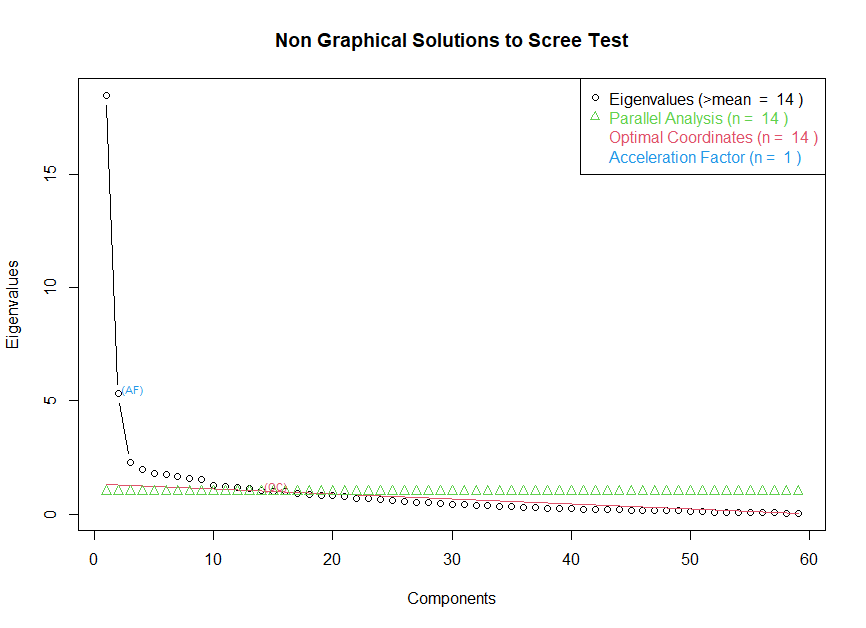

Supplement: Supplementary file 2 — Supplementary Material 2 [file 41687_2025_893_MOESM2_ESM.docx]
