# Supplementary material for: Participation and quality of life of Nepalese children with visual impairment in comparison with normally sighted peers: a cross sectional comparative study
Source: J Patient Rep Outcomes. 2025 Jun 5;9:64. doi: 10.1186/s41687-025-00893-2 (PMC12141697; doi:10.1186/s41687-025-00893-2)
Supplement: Supplementary file 3 — Supplementary Material 3 [file 41687_2025_893_MOESM3_ESM.docx]

**Supplementary table 1:** Distribution of responses over the response categories and factor loadings of the PAI-CY 7-12 (N=165)

| **Item No** | **Item description^a^** | **Distribution of responses over response categories^b^ (N)** | | | | **Missing (N)** | **Factor loadings** |
| --- | --- | --- | --- | --- | --- | --- | --- |
|  |  | **1** | **2** | **3** | **4** |  |  |
| **Section A: Play** | | | | | | | |
| A1 | Playing imaginary games | 116 | 28 | 21 | - | 0 | 0.47 |
| A2 | Participating in a game with rules | 112 | 28 | 23 | 2 | 0 | 0.59 |
| A3 | Keeping up with other children while playing | 117 | 21 | 27 | - | 0 | 0.67 |
| A4 | Playing in the playground | 123 | 21 | 20 | - | 1 | 0.61 |
| A5 | Playing inside the room | 135 | 16 | 14 | - | 0 | 0.43 |
| **Section B: Social contact** | | | | | | | |
| B1 | Contacting other children | 151 | 8 | 6 | - | 0 | 0.22 |
| B2 | Playing with other children without visual impairment | 113 | 20 | 31 | - | 1 | 0.74 |
| B3 | Playing outside/outdoors with friends | 116 | 21 | 27 | - | 1 | 0.77 |
| B4 | Playing at a friend’s house | 111 | 24 | 28 | 1 | 3 | 0.67 |
| B5 | Independently inviting a friend to play at your house | 113 | 16 | 12 | 1 | 3 | 0.51 |
| B6 | Participating in group activities | 89 | 30 | 45 | - | 3 | 0.80 |
| **Section C: Mobility** | | | | | | | |
| C1 | Cycling independently | 95 | 21 | 48 | 1 | 0 | 0.70 |
| C2 | Participating in high-speed activities | 85 | 32 | 47 | 1 | 0 | 0.79 |
| C3 | Independently and safely participating in traffic | 53 | 25 | 86 | 1 | 0 | 0.51 |
| C4 | Learning fixed routes | 77 | 24 | 63 | 1 | 0 | 0.72 |
| C5 | Finding way on unknown environment | 38 | 25 | 101 | 1 | 0 | 0.56 |
| C6 | Participating in traffic at night | 42 | 19 | 104 | - | 0 | 0.60 |
| **Section D: Leisure time** | | | | | | | |
| D1 | Reading books | 109 | 31 | 25 | - | 0 | 0.39 |
| D2 | Using social media | 96 | 21 | 48 | - | 0 | 0.61 |
| D3 | Playing games on the computer, tablet or phone (smartphone) | 101 | 18 | 46 | - | 0 | 0.73 |
| D4 | Watching films or TV | 114 | 15 | 36 | - | 0 | 0.63 |
| D5 | Going to a club/association independently | 60 | 24 | 81 | - | 0 | 0.64 |
| D6 | Participating at a club or association | 67 | 25 | 71 | 1 | 1 | 0.53 |
| D7 | Making music | 86 | 28 | 51 | - | 0 | 0.48 |
| **Section E: Communication** | | | | | | | |
| E1 | Telling parents what you want to say, while they understand what you mean | 138 | 21 | 5 | 1 | 0 | 0.22 |
| E2 | Asking questions to, for example, your teacher | 139 | 16 | 1 | 9 | 0 | 0.18 |
| E3 | Expressing your feelings | 132 | 19 | 14 | - | 0 | 0.35 |
| E4 | Telling your parents what you have done at school or at a friend’s house | 140 | 13 | 12 | - | 0 | 0.36 |
| E5 | Participating in a conversation | 137 | 17 | 11 | - | 0 | 0.29 |
| E6 | Asking for help from people you know | 146 | 12 | 7 | - | 0 | 0.21 |
| E7 | Asking for help from people you do not know | 43 | 34 | 88 | - | 0 | 0.36 |
| E8 | Indicating what you can or can’t see | 115 | 27 | 23 | - | 0 | 0.55 |
| E9 | Telling how other children are feeling | 71 | 23 | 70 | 1 | 0 | 0.50 |
| E10 | Determining how close you can stand to another person | 116 | 23 | 26 | - | 0 | 0.56 |
| E11 | Indicating or telling that you want to participate with other children in a group | 122 | 21 | 21 | - | 0 | 0.51 |
| E12 | Dealing with other children who bully | 81 | 35 | 49 | - | 0 | 0.58 |
| **Section F: School** | | | | | | | |
| F1 | Finding the way in school | 138 | 13 | 14 | - | 0 | 0.55 |
| F2 | Maintaining overview in the classroom | 138 | 13 | 13 | 1 | 0 | 0.54 |
| F3 | Keeping up with the pace of peers in class | 105 | 35 | 25 | - | 0 | 0.56 |
| F4 | Reading the digital board or school board | 66 | 33 | 64 | 1 | 1 | 0.75 |
| F5 | Finishing homework independently | 145 | 10 | 10 | - | 0 | 0.55 |
| F6 | Writing | 153 | 6 | 6 | - | 0 | 0.33 |
| F7 | *Reading braille* | 46 | 4 | 4 | 7 | 104 | - |
| F8 | Looking up information | 90 | 22 | 52 | - | 1 | 0.53 |
| F9 | Finding things in the closet or drawer at school | 112 | 24 | 28 | 1 | 0 | 0.69 |
| F10 | Collaborating with other children | 114 | 20 | 30 | 1 | 0 | 0.69 |
| F11 | Participating in physical education | 110 | 19 | 35 | 1 | 0 | 0.73 |
| F12 | Maintaining enough energy after school for ‘fun’ activities | 112 | 23 | 30 | - | 0 | 0.49 |
| **Section G: Self-reliance** | | | | | | | |
| G1 | Eating with fork and knife | 151 | 10 | 4 | - | 0 | 0.32 |
| G2 | Making a sandwich | 125 | 15 | 25 | - | 0 | 0.63 |
| G3 | *Brushing your teeth* | 165 | - | - | - | 0 | - |
| G4 | *Going to the restroom independently* | 158 | 5 | 1 | 1 | 0 | - |
| G5 | Taking a shower or bath independently | 130 | 13 | 22 | - | 0 | 0.37 |
| **Section H: Acceptance/self-consciousness** | | | | | | | |
| H1 | Telling others about your visual impairment | 129 | 19 | 17 | - | 0 | 0.40 |
| H2 | Accepting that one cannot do certain activities, because of visual impairment | 104 | 30 | 30 | 1 | 0 | 0.51 |
| H3 | Accepting that sometimes you make mistakes | 128 | 18 | 19 | - | 0 | 0.44 |
| H4 | Empathizing with others | 107 | 22 | 36 | - | 0 | 0.51 |
| H5 | Using (visual) aids | 121 | 11 | 32 | - | 1 | 0.56 |
| **Section I: Finances** | | | | | | | |
| I1 | Recognizing money | 114 | 16 | 35 | - | 0 | 0.61 |

**Supplementary table 2:** Distribution of responses over the response categories and factor loadings of the PAI-CY 13-17 (N=135)

| **Item No** | **Item description^a^** | **Distribution of responses over response categories^b^ (N)** | | | | **Missing (N)** | **Factor loadings^c^** | |
| --- | --- | --- | --- | --- | --- | --- | --- | --- |
|  |  | **1** | **2** | **3** | **4** |  | **Factor 1** | **Factor 2** |
| **Section A: Leisure time** | | | | | | | |  |
| A1 | Sporting | 70 | 24 | 38 | 3 | 0 | **0.64** |  |
| A2 | Keeping up with peers during play/sport | 62 | 18 | 53 | 1 | 1 | **0.77** |  |
| A3 | Using social media | 89 | 22 | 24 | - | 0 | **0.44** |  |
| A4 | Playing games on the computer, tablet or phone | 67 | 24 | 43 | - | 1 | **0.78** |  |
| A5 | Watching films or TV | 75 | 17 | 43 | - | 0 | **0.77** |  |
| A6 | Going to a club/association independently | 65 | 10 | 59 | - | 1 | **0.79** |  |
| A7 | Participating at a club or association | 86 | 16 | 32 | 1 | 0 | **0.55** | 0.35 |
| A8 | Making music | 98 | 13 | 24 | - | 0 | 0.29 | **0.29** |
| **Section B: Mobility** | | | | | | | |  |
| B1 | Cycling independently | 67 | 14 | 51 | 2 | 1 | **0.81** |  |
| B2 | Cycling someplace independently | 68 | 15 | 51 | 1 | 0 | **0.77** |  |
| B3 | Going to a friend in the neighborhood independently | 111 | 10 | 14 | - | 0 | **0.59** |  |
| B4 | Participating in traffic independently | 71 | 15 | 49 | - | 0 | **0.84** |  |
| B5 | Estimating speed | 76 | 13 | 46 | - | 0 | **0.76** |  |
| B6 | Using public transport independently | 61 | 19 | 54 | 1 | 0 | **0.77** |  |
| B7 | Learning fixed, new routes | 92 | 13 | 30 | - | 0 | **0.72** |  |
| B8 | Participating in traffic at night | 61 | 9 | 65 | - | 0 | **0.80** |  |
| **Section C: Social contacts** | | | | | | | |  |
| C1 | Contacting others | 111 | 7 | 16 | - | 1 | **0.51** |  |
| C2 | Participating in activities with peers without visual impairment | 64 | 23 | 47 | - | 1 | **0.75** |  |
| C3 | Participating in group activities | 65 | 13 | 56 | 1 | 0 | **0.80** |  |
| C4 | Going to shopping with friends | 111 | 8 | 15 | - | 1 | **0.53** |  |
| C5 | Going out with friends | 109 | 17 | 9 | - | 0 | **0.47** |  |
| C6 | Dating | 118 | 11 | 6 | - | 0 | 0.40 | **0.47** |
| C7 | Dealing with feelings of amorousness | 107 | 11 | 17 | - | 0 | 0.20 | **0.17** |
| **Section D: Communication** | | | | | | | |  |
| D1 | Expressing in words properly | 115 | 12 | 8 | - | 0 |  | **0.40** |
| D2 | Asking questions | 122 | 8 | 4 | 1 | 0 |  | **0.49** |
| D3 | Talking about feelings | 121 | 7 | 7 | - | 0 |  | **0.50** |
| D4 | *Participating actively in a conversation* | 131 | 4 | - | - | 0 |  |  |
| D5 | Asking for help from people you know | 129 | 4 | 1 | 1 | 0 |  | **0.44** |
| D6 | Asking for help from people you do not know | 61 | 26 | 48 | - | 0 | **0.46** | 0.21 |
| D7 | Estimating the emotions of others | 91 | 15 | 28 | 1 | 1 | **0.53** | 0.38 |
| D8 | Estimating the physical distance to others | 102 | 19 | 14 | - | 0 | **0.62** | 0.09 |
| D9 | Indicating that you want to participate in a group | 107 | 14 | 14 | - | 0 | **0.43** |  |
| D10 | Expressing your own opinion | 122 | 8 | 5 | - | 0 |  | **0.57** |
| D11 | Dealing with other children who bully | 96 | 11 | 28 | - | 0 | **0.55** |  |
| D12 | Recognizing other people | 98 | 18 | 19 | - | 0 | **0.58** |  |
| **Section E: School** | | | | | | | |  |
| E1 | Finding the way in school | 117 | 12 | 6 | - | 0 | **0.55** |  |
| E2 | Maintaining overview in the classroom | 91 | 14 | 29 | 1 | 0 | **0.67** |  |
| E3 | Keeping up with the pace of peers in class | 97 | 17 | 21 | - | 0 | **0.53** |  |
| E4 | Collaborating with others | 117 | 14 | 4 | - | 0 | **0.45** |  |
| E5 | Reading the digital board | 52 | 15 | 66 | 2 | 0 | **0.83** |  |
| E6 | Making homework independently | 117 | 13 | 5 | - | 0 | **0.36** |  |
| E7 | Looking up information | 98 | 18 | 19 | - | 0 | **0.62** |  |
| E8 | Maintaining enough energy after school for ‘fun’ activities | 114 | 11 | 10 | - | 0 | **0.46** |  |
| E9 | Choosing appropriate further education | 102 | 19 | 13 | - | 1 | 0.38 | **0.44** |
| E10 | Taking part in outside school activities | 106 | 13 | 16 | - | 0 | **0.52** | 0.37 |
| **Section F: Self-reliance** | | | | | | | |  |
| F1 | Cooking | 90 | 15 | 30 | - | 0 | **0.62** |  |
| F2 | Doing the dishes | 129 | 3 | 1 | - | 2 | **0.24** |  |
| F3 | Operating devices at home | 112 | 10 | 13 | - | 0 | **0.60** |  |
| F4 | Grocery shopping | 89 | 12 | 34 | - | 0 | **0.77** |  |
| F5 | Picking clothes independently | 104 | 11 | 20 | - | 0 | **0.58** |  |
| F6 | *Brushing your teeth independently* | 135 | - | - | - | 0 |  |  |
| F7 | Going to the restroom independently | 131 | 3 | 1 | - | 0 | 0.16 | **0.21** |
| F8 | *Showering/bathing independently* | 133 | 2 | - | - | 0 |  |  |
| F9 | *Styling hair* | 135 | - | - | - | 0 |  |  |
| F10 | *Dealing with menstruation* | 50 | 2 | 2 | - | 84 |  |  |
| F11 | Paying attention to facial care | 120 | 9 | 6 | - | 0 | **0.27** | 0.14 |
| **Section G: Acceptance/self-consciousness** | | | | | | | |  |
| G1 | Telling about visual impairment | 114 | 10 | 9 | - | 2 | 0.18 | **0.11** |
| G2 | Empathizing with others | 118 | 9 | 8 | - | 0 |  | **0.55** |
| G3 | Accepting that one cannot do certain things/activities | 114 | 11 | 10 | - | 0 | 0.36 | **0.47** |
| G4 | Accepting that one sometimes makes mistakes | 124 | 7 | 4 | - | 0 |  | **0.43** |
| G5 | Doing activities without getting fatigued | 124 | 5 | 6 | - | 0 |  | **0.57** |
| G6 | Dividing energy during the day | 114 | 13 | 8 | - | 0 | 0.37 | **0.53** |
| **Section H: Finances** | | | | | | | |  |
| H1 | Paying independently | 94 | 7 | 29 | 1 | 0 | **0.71** |  |

^a^ Item description is abbreviated and not an official translation; italic items were deleted before the principal component analyses
^b^ Response categories: 1: Not difficult; 2: Slightly difficult; 3: Very difficult; 4: Impossible
^c^ Bold loadings indicate to which factor an item belongs; factor 1: physical functioning; factor 2: Psychosocial functioning

Supplementary Table 3: Distribution of responses over response categories of PedEyeQ (5-11,12-17) and LVP-FVQ II

|  | Items with Domains  **PedEyeQ (5-11)** N= 93 | Distribution of Responses over categories | Missing responses |
| --- | --- | --- | --- |
|  | **Section A:** Functional vision | 0 1 2 |  |
| A1 | Do your eyes make it hard to learn | 38 38 17 | 0 |
| A2 | Do you have a hard time seeing? | 52 26 15 | 0 |
| A3 | Do you have to do things differently than other people because of your eyes? | 31 34 28 | 0 |
| A4 | Do your eyes make it hard to concentrate? | 32 26 35 | 0 |
| A5 | Do your eyes make it hard to do certain things? | 36 29 28 | 0 |
| A6 | Do you have trouble reading close-up? | 36 11 46 | 0 |
| A7 | Do you have to do certain things to help you see better? | 66 11 16 |  |
| A8 | Is it hard to see the board at school? | 58 12 23 | 0 |
| A9 | Do you run into things because of your eyes? | 22 47 24 | 0 |
| A10 | Do your eyes get tired easily? | 19 17 57 | 0 |
|  | **Section B: Bothered by eye/vision** |  |  |
| B1 | Does it bother you because your eyes make it hard to learn? | 31 27 35 | 0 |
| B2 | Does it bother you because your eyes make it hard to play sports? | 25 22 42 | 0 |
| B3 | Does it bother you because you have a hard time seeing? | 34 20 39 | 0 |
| B4 | Does it bother you that you can't do certain things because of your eyes? | 30 19 44 | 0 |
| B5 | Does it bother you because your eyes make it hard to do certain things? | 27 22 44 | 0 |
| B6 | Does it bother you because it's hard to see the board at school? | 32 21 40 | 0 |
| B7 | Does it bother you because it's hard to see steps when you walk? | 34 19 40 | 0 |
| B8 | Does it bother you that bright light makes it hard to do things outside? | 35 14 44 | 0 |
| B9 | Is taking care of your eye condition hard for you? | 26 13 54 | 0 |
| B10 | Does it bother you because your eyes hurt? | 31 15 47 | 0 |
|  | **Section C: Social** |  |  |
| C1 | Does it bother you that it's hard to play/interact with others because of your eyes? | 26 21 46 | 0 |
| C2 | Does your eye condition cause problems in your family? | 19 17 57 | 0 |
| C3 | Are you shy because of your eyes? | 10 15 68 | 0 |
| C4 | Do other people get frustrated with you because of your eyes? | 19 20 54 | 0 |
| C5 | Do you get teased because of your eyes? | 11 17 65 | 0 |
| C6 | Do you worry about getting hurt because of your eyes? | 23 13 57 | 0 |
| C7 | Do you worry about your eyes getting worse? | 36 8 49 | 0 |
| C8 | Do you worry about what other people think about you because of your eyes? | 28 9 56 | 0 |
| C9 | Do you worry about getting teased because of your eyes? | 27 9 57 | 0 |
| C10 | Do you worry about your eyes? | 26 12 55 | 0 |
|  | **Section D: Frustration/worry** |  | 0 |
| D1 | Are you bothered by the things you have to do to make your eyes better? | 29 7 57 | 0 |
| D2 | Do your eyes make you feel unsure of yourself? | 33 18 42 | 0 |
| D3 | Are you frustrated because your eyes aren't getting better? | 30 14 49 | 0 |
| D4 | Does it bother you when other people say things or ask questions about your eyes? | 27 14 52 | 0 |
| D5 | Do you feel "different" because of your eyes? | 26 15 52 | 0 |
| D6 | Does it bother you that you get extra attention because of your eyes? | 15 10 68 | 0 |
| D7 | Do you feel left out because of your eyes? | 11 11 71 | 0 |
| D8 | Do you worry that your eyes will make it hard to do things when you're older? | 21 11 61 | 0 |
| D9 | Do you worry about not being able to do things because of your eyes? | 23 16 54 | 0 |
| D10 | 10. Do you worry about what you might have to do to make your eyes better? | 35 15 43 | 0 |
|  | **PedEyeQ (12-17)** N=106 |  |  |
|  | **Section A: Functional vision** |  |  |
| A1 | Do your eyes make it hard to learn? | 29 55 12 | 0 |
| A2 | Do your eyes make it hard to play sports? | 48 39 19 | 0 |
| A3 | Do you have a hard time seeing? | 69 22 15 | 0 |
| A4 | Do you need special help at school because of your eyes? | 75 25 6 | 0 |
| A5 | Do you need help with certain things because of your eyes? | 59 37 10 | 0 |
| A6 | Do you have to do things differently than other people because of your eyes? | 51 39 16 | 0 |
| A7 | Do your eyes make it hard to concentrate? | 37 37 32 | 0 |
| A8 | Is it hard to see the board at school? | 86 9 11 | 0 |
| A9 | Is it hard to see steps when you walk? | 44 18 14 | 0 |
| A10 | Do you run into things because of your eyes? | 26 66 14 | 0 |
|  | **Section B: Bothered by eye/vision** |  |  |
| B1 | Does it bother you that you need help with certain things because of your eyes? | 18 41 47 | 0 |
| B2 | Does it bother you to have to do things differently because of your eyes? | 20 31 55 | 0 |
| B3 | Does it bother you because your eyes make it hard to learn? | 37 33 36 | 0 |
| B4 | Does it bother you because your eyes make it hard to play sports? | 42 28 36 | 0 |
| B5 | Does it bother you because you have a hard time seeing? | 58 26 22 | 0 |
| B6 | Does it bother you to need special help at school because of your eyes? | 25 40 41 | 0 |
| B7 | Does it bother you because your eyes make it hard to concentrate? | 34 31 41 | 0 |
| B8 | Does it bother you because your eyes make it hard to do certain things? | 41 33 32 | 0 |
| B9 | Does it bother you because it's hard to see the board at school? | 67 24 15 | 0 |
| B10 | Are you bothered by the things you have to do to make your eyes better? | 54 24 28 | 0 |
|  | **Section C: Social** |  |  |
| C1 | Are you bothered by how your eyes look? | 33 18 55 | 0 |
| C2 | Do your eyes make you feel unsure of yourself? | 45 32 29 | 0 |
| C3 | Does it bother you when other people say things or ask questions about your eyes? | 32 22 52 | 0 |
| C4 | Do you feel "different" because of your eyes? | 43 33 30 | 0 |
| C5 | Does it bother you that you get extra attention because of your eyes? | 29 26 51 | 0 |
| C6 | Does it bother you when people look/stare at you because of your eyes? | 41 28 37 | 0 |
| C7 | Are you shy because of your eyes? | 23 11 72 | 0 |
| C8 | Do you get teased because of your eyes? | 8 17 81 | 0 |
| C9 | Do you worry about what other people think about you because of your eyes? | 30 21 55 | 0 |
| C10 | Do you worry about getting teased because of your eyes? | 27 21 58 | 0 |
|  | **Section D: Frustration/worry** |  |  |
| D1 | Do you hate going to the eye doctor? | 6 7 93 | 0 |
| D2 | Is taking care of your eye condition hard for you? | 16 15 75 | 0 |
| D3 | Are you frustrated because your eyes aren't getting better? | 47 25 34 | 0 |
| D4 | Do you worry about getting hurt because of your eyes? | 31 26 49 | 0 |
| D5 | Do you worry about your eyes getting worse? | 54 20 32 | 0 |
| D6 | Do you worry that your eyes will make it hard to do things when you're older? | 52 23 31 | 0 |
| D7 | Do you worry about not being able to do things because of your eyes? | 41 29 36 | 0 |
| D8 | Do you worry about your eyes? | 52 26 28 | 0 |
| D9 | Do you worry about what you might have to do to make your eyes better? | 67 21 18 | 0 |
|  |  | **Response categories** |  |
|  | **LVP-FVQ II** N=300 | **1 2 3** |  |
| A1 | Reading the bus numbers | 133 50 116 | 0 |
| A2 | Finding out the next line while reading | 167 45 87 | 0 |
| A3 | Locating dropped objects | 181 55 63 | 0 |
| A4 | Threading a needle | 95 25 179 | 0 |
| A5 | Locating ball while playing in the daytime | 185 29 85 | 0 |
| A6 | Copying from your friend’s notebook | 167 35 97 | 0 |
| A7 | Reading the rates on items (e.g., chips packets, biscuit packets) | 134 33 132 | 0 |
| A8 | Reading the phone numbers on mobile phone | 173 28 98 | 0 |
| A9 | Watching TV | 170 47 82 | 0 |
| A10 | Seeing time in your wrist watch | 159 26 114 | 0 |
| A11 | 15. Reading from a computer screen | 126 40 133 | 0 |
| A12 | Copying the small letters from the board | 99 32 168 | 0 |
| A13 | Walking on uneven ground | 166 41 92 | 0 |
| A14 | Reading shop names | 149 36 114 | 0 |
| A15 | Seeing animals in zoo | 184 27 88 | 0 |
| A16 | Identifying dirt, stains on your own clothes | 185 27 87 | 0 |
| A17 | Reading your books at near | 198 19 82 | 0 |
| A18 | Seeing your friend in the playground while playing | 181 42 76 | 0 |
| A19 | Watching a movie at the theatre | 175 36 88 | 0 |
| A20 | Selecting a song using iPod | 188 20 91 | 0 |
| A21 | Playing video games | 170 30 99 | 0 |
| A22 | Seeing the numbers and markings on the scale | 155 24 120 | 0 |
| A23 | Your vision compared to your normally-sighted friend | 108 99 92 | 0 |

PedEyeQ response categories; 1: All the times, 2: Sometimes, 3: Never LVP-FVQ response categories; 1: No difficulty, 2: Some difficulty, 3: A lot of difficulty
